# Neuropsychiatric symptoms and progression to pathologically confirmed Alzheimer’s disease

**DOI:** 10.1093/brain/awaf156

**Published:** 2025-04-25

**Authors:** Sergio F Sharif, Dylan X Guan, Tamara S Bodnar, Jeffery T Joseph, Henrik Zetterberg, Eric E Smith, Zahinoor Ismail

**Affiliations:** Cumming School of Medicine, University of Calgary, Calgary, AB, Canada T2N 1N4; Cumming School of Medicine, University of Calgary, Calgary, AB, Canada T2N 1N4; Hotchkiss Brain Institute, University of Calgary, Calgary, AB, Canada T2N 4Z6; Department of Biological Sciences, University of Calgary, Calgary, AB, Canada T2N 1N4; Hotchkiss Brain Institute, University of Calgary, Calgary, AB, Canada T2N 4Z6; Department of Pathology and Laboratory Medicine, University of Calgary, Calgary, AB, Canada T2N 4Z6; Department of Psychiatry and Neurochemistry, Institute of Neuroscience and Physiology, the Sahlgrenska Academy at the University of Gothenburg, Mölndal 431 39, Sweden; Clinical Neurochemistry Laboratory, Sahlgrenska University Hospital, Mölndal 431 39, Sweden; Department of Neurodegenerative Disease, UCL Institute of Neurology, London WC1N 3BG, UK; UK Dementia Research Institute at UCL, London NW1 3BT, UK; Hong Kong Center for Neurodegenerative Diseases, Clear Water Bay, Hong Kong, China; Wisconsin Alzheimer’s Disease Research Center, University of Wisconsin School of Medicine and Public Health, University of Wisconsin-Madison, Madison, WI 53792, USA; Hotchkiss Brain Institute, University of Calgary, Calgary, AB, Canada T2N 4Z6; Department of Community Health Sciences, University of Calgary, Calgary, AB, Canada T2N 4Z6; Department of Clinical Neurosciences, University of Calgary, Calgary, AB, Canada T2N 4Z6; Hotchkiss Brain Institute, University of Calgary, Calgary, AB, Canada T2N 4Z6; Department of Pathology and Laboratory Medicine, University of Calgary, Calgary, AB, Canada T2N 4Z6; Department of Community Health Sciences, University of Calgary, Calgary, AB, Canada T2N 4Z6; Department of Clinical Neurosciences, University of Calgary, Calgary, AB, Canada T2N 4Z6; O’Brien Institute for Public Health, University of Calgary, Calgary, AB, Canada T2N 4Z6; Department of Psychiatry, University of Calgary, Calgary, AB, Canada T2N 4Z6; Clinical and Biomedical Sciences, Faculty of Health and Life Sciences, University of Exeter, Exeter EX1 2HZ, UK

**Keywords:** mild behavioural impairment, dementia, TDP-43, neuropsychiatry, neuropathology, cognitive decline

## Abstract

Whether or not neuropsychiatric symptoms (NPS) in advance of dementia are associated with Alzheimer’s disease (AD) and/or other neurodegenerative dementias remains to be determined. The mild behavioural impairment (MBI) construct selects persons with NPS that are later-life emergent and persistent to identify a high-risk group for cognitive decline and incident dementia. Here, in older adults without dementia at baseline, we examined whether post-mortem AD and other neurodegenerative pathologies were associated with MBI in the 5 years before death.

National Alzheimer’s Coordinating Center study autopsy participants (*n* = 1016, 82.6 years of age, 48.7% female, 60% normal cognition) were included in the analyses. Using the Neuropsychiatric Inventory-Questionnaire, MBI+ status was operationalized as NPS persistence at more than two-thirds of pre-dementia study visits; otherwise, status was non-MBI NPS. The presence of AD, Lewy body disease (LBD) and transactive response DNA-binding protein 43 neuropathological changes was determined using published guidelines. Adjusted multinomial logistic regressions modelled pathology–NPS status associations. Adjusted Cox proportional hazards regressions modelled hazard for AD dementia at each NPS status level, including interaction terms with cognitive status and each co-pathology.

AD+ individuals (51.4%) were 88.4% more likely to be MBI+ ∼5 years prior than AD− individuals [odds ratio: 1.88, 95% confidence interval (CI): 1.29–2.75, *P* < 0.01]; however, the likelihood of having non-MBI NPS was not different (odds ratio: 1.22, CI: 0.90–1.66, *P* = 0.20). No significant associations were seen for LBD pathology, even among AD+ participants. There were no significant differences in the levels of LBD or transactive response DNA-binding protein 43 in those with MBI in comparison to no MBI. Among MBI progressors to dementia (*n* = 106), 33.0% were solely AD+, 18.9% were mixed AD+ and LBD+, and 11.3% had all three pathologies. For all those with MBI (including dementia non-progressors), of persons with LBD, 83.4% were comorbid with AD. In the survival analysis, MBI+ individuals had a 2.03-fold greater progression rate to AD dementia than those without NPS (CI: 1.60–2.57, *P* < 0.01). The progression rate was higher in mild cognitive impairment , but the effect of MBI on progression was greater in normal cognition (hazard ratio: 3.05, CI: 1.37–6.80, *P* < 0.01) versus mild cognitive impairment (hazard ratio: 1.93, CI: 1.51–2.47, *P* < 0.01). Limbic LBD appeared also to moderate the association between MBI and incident AD (limbic LBD+ hazard ratio: 4.64, CI: 2.05–10.50, *P* < 0.001; limbic LBD− hazard ratio: 1.87, CI: 1.46–2.40, *P* < 0.001).

Antecedent MBI was strongly associated with AD pathology but not with other neurodegenerative dementias. Inclusion of MBI in research and clinical frameworks for dementia might aid in identification of early stages of neurodegenerative disease, which might be helpful for selecting patients for treatment with AD-modifying drugs.

## Teaser

Psychiatric symptoms emerging later in life may be early signs of neurodegenerative disease. Sharif *et al.* show that older adults who met criteria for mild behavioural impairment progressed more rapidly to Alzheimer’s disease dementia, and were enriched for Alzheimer’s disease pathology at autopsy.

## Introduction

Alzheimer’s disease (AD) is the most common cause of dementia, with neuropathological assessment serving as the gold standard for diagnosis.^1^ Recent evidence points to neuropsychiatric symptoms (NPS) as potential preclinical/prodromal aspects of AD dementia.^2-9^ However, it is unclear whether inclusion of NPS in staging of individuals along the AD continuum is of benefit.^10,11^ Whether specific NPS have distinct pathological substrates or are common across multiple diseases also remains uncertain.^10,12,13^ Without consideration of the natural history of symptoms, research on NPS might fail to distinguish between long-standing behaviours or personality differences related to psychiatric disorders and later-life-onset psychiatric symptoms that are prodromal to neurodegenerative disease.^4,14,15^

To address this issue, the Neuropsychiatric Syndromes Professional Interest Area of the Alzheimer’s Association International Society to Advance Alzheimer's Research and Treatment developed the mild behavioural impairment (MBI) construct. The MBI syndromic criteria were created to incorporate NPS better into dementia risk assessment, leveraging the higher risk associated with NPS that emerge *de novo* in later life and that persist for ≥6 months.^2^ This construct has shed light on the utility of NPS meeting MBI criteria for prognosticating cognitive decline and incident dementia. Studies of MBI have demonstrated: (i) poorer cognitive performance and faster cognitive decline; (ii) greater progression rates from normal cognition (NC) and subjective cognitive decline to mild cognitive impairment (MCI); and (iii) greater progression rates from MCI to dementia, with lower reversion rates from MCI to NC, relative to non-MBI comparator groups.^16-22^

MBI has demonstrated associations with many AD markers.^23-27^ Furthermore, in line with the National Institute on Aging–Alzheimer’s Association (NIA-AA) Amyloid-Tau-Neurodegeneration (ATN) Research Framework for AD,^28^ several studies have demonstrated associations between MBI and biomarkers related to the AD framework, such as amyloid-β and tau, in addition to the AD-associated *APOE* ɛ4 allele.^25,29-34^ Thus, current evidence suggests that of persons with NPS, MBI criteria select for a subset who are at high risk for cognitive decline and incident dementia and who might already be on the AD continuum.

With respect to non-AD pathologies, a study in cognitively unimpaired participants found an association between an approximation of MBI and greater progression rate to clinically diagnosed and neuropathologically confirmed AD, although co-pathologies were not reported.^23^ A growing body of evidence points to the additive and synergistic effect of multiple pathologies in the development of dementia symptoms.^12,35-37^ However, more research is needed, especially regarding the differential influence of Lewy body disease (LBD) and/or transactive response DNA-binding protein 43 (TDP-43) pathology on NPS presentations in those with AD. Furthermore, data with preclinical and prodromal behavioural symptoms are sparse. Owing to the potential diagnostic and prognostic utility of MBI, additional gold standard neuropathological assessments are required, using current approaches to operationalizing MBI and incorporating additional pathologies in the modelling.

Here, we examined the links between AD, post-mortem co-pathologies and MBI. Specifically, we tested whether AD neuropathological change (ADNC), LBD pathology and/or TDP-43 inclusions were associated with a greater likelihood of preceding MBI, in the 5 years before death. We also tested whether baseline MBI was predictive of a greater progression rate to AD dementia, the potential moderation of cognitive status and/or co-pathology for incident AD dementia, and whether there were group differences in participant pathological profiles. We hypothesized that those with MBI would progress more rapidly to neuropathologically confirmed AD, thus identifying a group enriched for AD pathology post-mortem.

## Materials and methods

### Source population

Data were obtained from the National Alzheimer’s Coordinating Center (NACC) database (https://naccdata.org), with a May 2022 data freeze. NACC was established by the National Institute on Aging and consists of multiple institute-funded Alzheimer’s Disease Research Centers recruiting and collecting data on participants with cognitive functions ranging from normal to dementia. The NACC Uniform Data Set is a large longitudinal dataset including demographic and standardized clinical data collected approximately annually. All test centres administered standardized forms, and informed consent was collected from all participants and their informants. All protocols were approved by the University of Washington institute review board. Detailed information on the cohort and neuropsychological battery of tests included in the Uniform Data Set are described elsewhere.^38-40^ Data from the NACC Neuropathology Data Set were collected according to a standardized Neuropathology Form and Coding Guidebook. In 2014, the Neuropathology Form version 10 was revised to NIA-AA guidelines,^41^ including updated classification of ADNC and incorporation of a wider range of neuropathological variables.^42^ Since the time of these analyses, a newer dataset has been released; however, the data-collection methods have not changed substantially.

### Participants

All participants (*n* = 1016) were in NACC and had data available in the Neuropathology Data Set with form version 10 or later.

**Figure 1:**
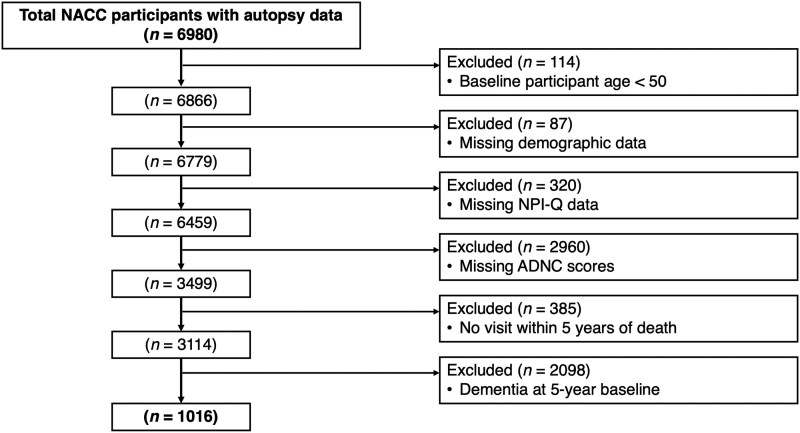
**Flow diagram for participant inclusion and exclusion criteria.** ADNC = Alzheimer's disease neuropathologic change; NACC = National Alzheimer’s Coordinating Center; NPI-Q = Neuropsychiatric Inventory-Questionnaire.

Figure 1 outlines the participant inclusion and exclusion criteria. To distinguish between later-life, *de novo* NPS with symptomatology attributable to longstanding neurological and/or psychiatric conditions, participants with a history of chronic and/or recurrent psychiatric disorders (e.g. depression, schizophrenia, bipolar disorder) and neurodevelopmental/neurological disorders (e.g. Down’s syndrome, autism, Huntington’s disease) were excluded. All available participant NPS data were used from pre-dementia visits. To maximize sample size, individuals with both NC and MCI were included (all analyses controlled for cognitive status).

### Neuropsychiatric symptom status

NPS severity was derived from the Neuropsychiatric Inventory-Questionnaire (NPI-Q)^43^ using a published algorithm,^44,45^ mapping 10 items onto the following five domains of MBI: (i) decreased motivation (apathy/indifference); (ii) emotional dysregulation (depression/dysphoria, anxiety, elation/euphoria); (iii) impulse dyscontrol (agitation/aggression, irritability/lability, aberrant motor behaviour); (iv) social inappropriateness (disinhibition); and (v) abnormal perception or thoughts (delusions and hallucinations). MBI domain scores were calculated by summing the scores of each corresponding NPI-Q domain item. MBI+ status was determined based on the most recently validated operational definition of MBI,^46^ with symptom persistence operationalized by the presence of NPS at more than two-thirds of study visits preceding either onset of dementia or death (whichever occurred first) and *de novo* symptom emergence determined by the absence of any previous psychiatric conditions. If NPS did not meet MBI criteria (i.e. impersistent NPS and/or occurring in the context of a previous psychiatric diagnosis), status was non-MBI NPS, and if no NPS were present, status was no NPS.

### Neuropathology

Participant pathology was derived from the Neuropathological Data Set, dichotomized by the presence/absence of each proteinopathy (AD, LBD and TDP-43). AD pathology was scored using the semi-quantitative ABC criteria, which account for Thal staging (criterion A), Braak staging (criterion B) and Consortium to Establish a Registry for Alzheimer’s Disease (CERAD) staging (criterion C). These criteria are then used to rate the likelihood of ADNC, resulting in a scale described as ‘Not’, ‘Low’, ‘Intermediate’ or ‘High’. AD status was derived using the NIA-AA recommendations for AD neuropathological assessment, with scores of ‘Intermediate’ or ‘High’ probability ADNC conferring AD+ status.^41^ For LBD pathology, participants were dichotomized based on the presence/absence of alpha-synuclein immunoreactivity anywhere in the brain. LBD pathology was assessed following a modified DLB Consortium classification^47^: brainstem-predominant, limbic (transitional), neocortical (diffuse), amygdala-predominant or olfactory bulb LBD. For TDP-43 pathology, participants were dichotomized based on the presence/absence of TDP-43-immunoreactive inclusions in any of the following regions: spinal cord, amygdala, hippocampus, entorhinal/inferior temporal cortex or neocortex.

### Statistical analysis

Following a case–control approach, a multinomial logistic regression was used to model the association between the presence of each pathology at autopsy (predictor) and NPS status out to 5 years before death (outcome), controlling for the presence of co-pathologies. To examine the potentially differing contributions of co-pathologies to MBI in AD+ individuals, another multinomial logistic regression was run between each co-pathology (i.e. LBD, TDP-43) as a predictor and NPS status as the outcome. The same model was run in AD− individuals to test for any associations between co-pathologies in those with low levels of ADNC. All models adjusted for age, sex, education, cognitive status (NC versus MCI), time to death, and *APOE* ɛ4 carrier status.

To determine whether MBI was associated with a greater progression rate to AD dementia (clinical diagnosis of dementia, AD+ at autopsy), Cox proportional hazards regression modelled the association between baseline NPS status and incident AD dementia within 5 years. NPS and/or cognitive status were time-varying covariates. All models adjusted for age, sex, education, cognitive status, time to death, and *APOE* ɛ4 carrier status, with interaction term NPS × Cognitive status (i.e. NC versus MCI).

In an exploratory analysis, the proportion of participant pathologies was tabulated and stratified by NPS status. An adjusted multinomial logistic regression modelling baseline NPS status (predictor) and each combination of pathologies (outcome) was also used, with the no pathology group (i.e. AD−, LBD−, TDP−) serving as the reference. The model was adjusted for age, sex, education, cognitive status, time to death, and *APOE* ɛ4 carrier status.

In a further exploratory analysis, interactions between NPS and proteinopathy status (i.e. LBD and TDP-43 pathology) were examined in a Cox proportional hazards model for baseline NPS status and AD dementia within 5 years, adjusted for age, sex, education, cognitive status, time to death, and *APOE* ɛ4 carrier status. To explore region-specific associations for LBD pathology further, the same analyses were repeated with an LBD variable stratified by the different regions reported for immunoreactivity [i.e. brainstem-predominant, limbic (transitional), neocortical (diffuse), amygdala-predominant or olfactory bulb].

Statistical analyses were performed in R v.4.3.0 using the *survival* package v.3.5-5 for Cox proportional hazards regression models and *ggplot2* v.3.4.2 and *survminer* v.0.4.9 packages for Kaplan–Meier curves and forest plots of hazard ratio. Assumptions for proportional hazards were assessed using the cox.zph() function from the survival package. Multinomial models were run using the *nnet* package v.7.3-18. Statistical significance was considered at a *P*-value of <0.05.

## Results

### Descriptive statistics

**Figure 2:**
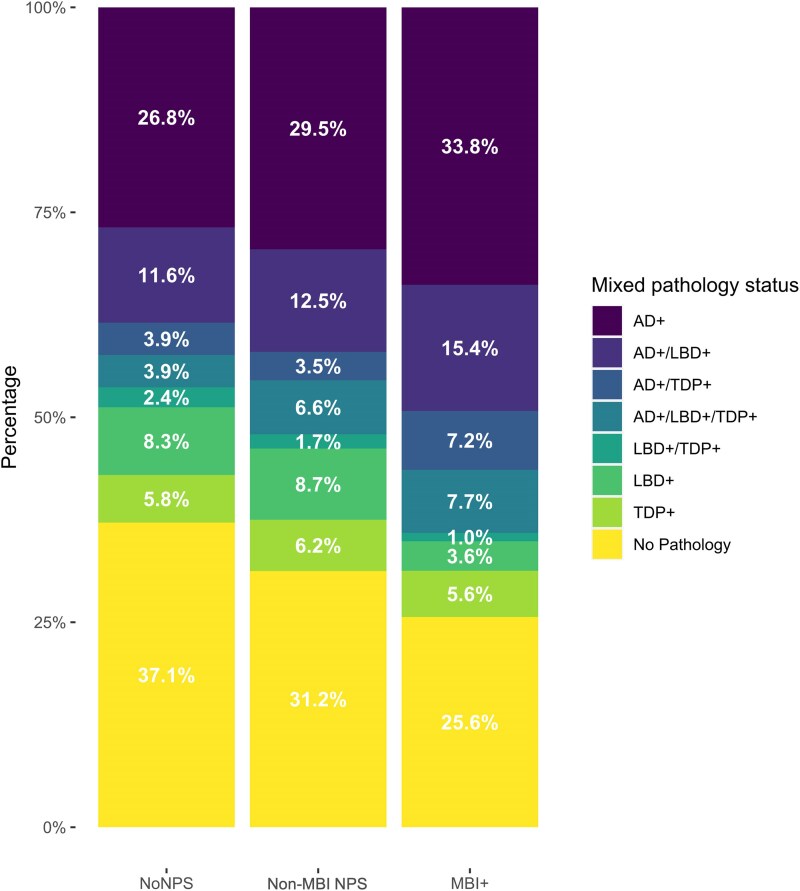
**Proportions of participants with AD, LBD and TDP-43 pathology, by NPS status.** AD = Alzheimer’s disease; LBD = Lewy body; MBI = mild behavioural impairment; NPS = neuropsychiatric symptoms.

The demographics of participants are summarized in Table 1. The proportion of pathologies, stratified by NPS status, are summarized in Fig. 2 and Table 2. Among those who progressed to dementia, NPS were assessed annually over an average of 2.72 ± 1.39 visits before the onset of dementia. Among those with MBI (*n* = 195), 33.8% were solely AD+, 15.4% were mixed AD+/LBD+, and 7.7% had AD, LBD and TDP-43 pathology. In the non-MBI NPS group, 29.5% were AD+, whereas 12.5% were AD+/LBD+, and 6.6% were AD+/LBD+/TDP+. For those with MBI, of persons with LBD, 83.4% were comorbid with AD. For non-MBI NPS, 64.8% had comorbid AD, and for no NPS it was 59.3%. Among those with MBI who progressed to dementia (*n* = 106), 33.0% were solely AD+, 18.9% were mixed AD+/LBD+, and 11.3% had AD, LBD and TDP-43 pathology. In the non-MBI NPS group (*n* = 125), 33.6% were AD+, whereas 16.8% were AD+/LBD+, and 11.2% were AD+/LBD+/TDP+.

**Table 1:** Participant characteristics, stratified by NPS status

	No NPS (*n* = 533)	Non-MBI NPS(*n* = 288)	MBI+ (*n* = 195)	Total(*n* = 1016)	*P-*value
Age	–	–	–	–	<0.001^a^
Mean (SD)	83.932 (8.365)	81.309 (8.775)	81.077 (10.058)	82.641 (8.924)	
**Sex**	–	–	–	–	0.014^b^
Male	255 (47.8%)	149 (51.7%)	117 (60.0%)	521 (51.3%)	
Female	278 (52.2%)	139 (48.3%)	78 (40.0%)	495 (48.7%)	
**Education**	–	–	–	–	0.222^a^
Mean (SD)	16.131 (2.996)	15.878 (2.860)	15.738 (3.049)	15.984 (2.970)	
**Cognitive Status**	–	–	–	–	<0.001^b^
NC	384 (72.0%)	161 (55.9%)	60 (30.8%)	605 (59.5%)	
MCI	149 (28.0%)	127 (44.1%)	135 (69.2%)	411 (40.5%)	
**AD status**	–	–	–	–	<0.001^b^
AD−	286 (53.7%)	138 (47.9%)	70 (35.9%)	494 (48.6%)	
AD+	247 (46.3%)	150 (52.1%)	125 (64.1%)	522 (51.4%)	
**LBD status**	–	–	–	–	0.608^b^
LBD−	393 (73.7%)	203 (70.5%)	141 (72.3%)	737 (72.5%)	
LBD+	140 (26.3%)	85 (29.5%)	54 (27.7%)	279 (27.5%)	
**TDP-43 status**	–	–	–	–	0.236^b^
TDP-43−	447 (83.9%)	236 (81.9%)	153 (78.5%)	836 (82.3%)	
TDP-43+	86 (16.1%)	52 (18.1%)	42 (21.5%)	180 (17.7%)	

AD+/− = Alzheimer’s disease present/absent; LBD+/− = Lewy body disease present/absent; MBI = mild behavioural impairment; MCI = mild cognitive impairment; NC = normal cognition; NPS = neuropsychiatric symptoms; SD = standard deviation; TDP-43+/− = transactive response DNA-binding protein 43 present/absent.

^a^Linear model ANOVA.

^b^Pearson’s χ^2^ test.

**Table 2:** Descriptive table of participant pathologies, stratified by NPS status

Pathological profile	No NPS(*n* = 533)	Non-MBI NPS(*n* = 288)	MBI+ (*n* = 195)	Total(*n* = 1016)
AD−LBD−TDP−	198 (37.1%)	90 (31.2%)	50 (25.6%)	338 (33.3%)
AD+	143 (26.8%)	85 (29.5%)	66 (33.8%)	294 (28.9%)
TDP+	31 (5.8%)	18 (6.2%)	11 (5.6%)	60 (5.9%)
LBD+	44 (8.3%)	25 (8.7%)	7 (3.6%)	76 (7.5%)
LBD+TDP+	13 (2.4%)	5 (1.7%)	2 (1.0%)	20 (2.0%)
AD+TDP+	21 (3.9%)	10 (3.5%)	14 (7.2%)	45 (4.4%)
AD+LBD+	62 (11.6%)	36 (12.5%)	30 (15.4%)	128 (12.6%)
AD+LBD+TDP+	21 (3.9%)	19 (6.6%)	15 (7.7%)	55 (5.4%)

AD+/− = Alzheimer’s disease present/absent; LBD+/− = Lewy body disease present/absent; MBI = mild behavioural impairment; NPS = neuropsychiatric symptoms; TDP-43+/− = transactive response DNA-binding protein 43 present/absent.

### Case–control analysis

In comparison to AD− individuals, AD+ individuals were 88% more likely to have MBI+ status in the 5 years preceding death [odds ratio: 1.88, 95% confidence interval (CI): 1.29–2.75, *P* < 0.001], but not more likely to have non-MBI NPS status (odds ratio: 1.22, CI: 0.90–1.66, *P* = 0.20). Neither LBD nor TDP-43 pathology was associated with a greater likelihood of having either MBI or non-MBI NPS in advance of dementia. In the sub-analysis of AD+ individuals, neither LBD nor TDP-43 co-pathologies showed any associations with NPS status; the same held true among AD− individuals.

### Survival analysis

**Figure 3:**
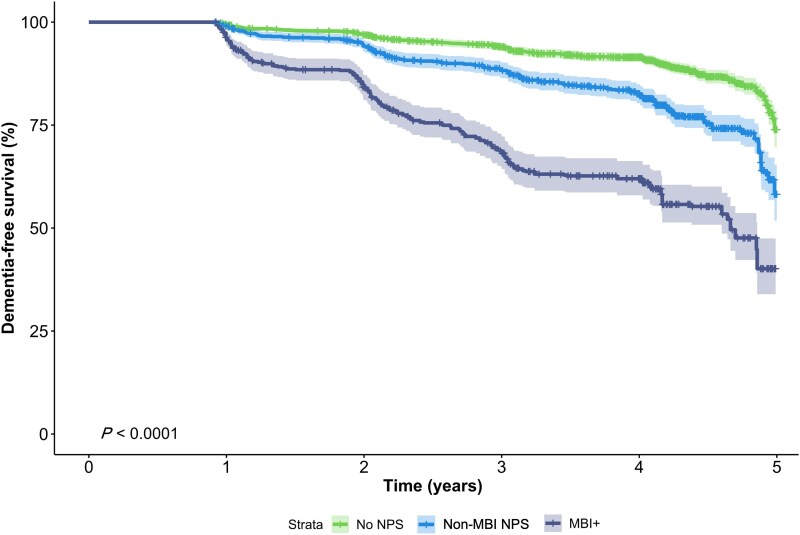
**Kaplan–Meier curve for 5-year progression to AD dementia, stratified by NPS status and results of Cox proportional hazards regression for progression to AD dementia.** All models adjusted for age, sex, education, cognitive status, time to death and *APOE* ɛ4 carrier status. AD = Alzheimer’s disease; *APOE* = apolipoprotein E; MBI = mild behavioural impairment; NPS= neuropsychiatric symptoms. See also Table 3.

Survival analysis revealed that MBI+ individuals had a 2.03-fold greater progression rate to AD dementia than those with no NPS (CI: 1.60–2.57, *P* < 0.001). Those with non-MBI NPS did not progress to the outcome any faster than the reference group (Fig. 3 and Table 3). For the interaction analysis, cognitive status did not moderate the relationship between MBI and AD dementia (*P* = 0.284), but the effect of MBI on progression was greater in NC (hazard ratio: 3.05, CI: 1.37–6.80, *P* = 0.006) versus MCI participants (hazard ratio: 1.93, CI: 1.51–2.47, *P* < 0.001).

**Table 3:** Results of Cox proportional hazards regression for progression to AD dementia

Characteristic	Subgroup	*n*	Hazard ratio	95% CI		*P*-value
				Lower	Upper	
NPS group	Non-MBI NPS	1126	1.027	0.800	1.319	0.8348
	MBI	572	2.027	1.599	2.569	<0.001*
Cognitive status	MCI	1418	14.908	10.617	20.933	<0.001*
Age of death	–	4515	0.965	0.954	0.976	<0.001*
Sex	Male	2244	1.081	0.885	1.321	0.444
Education	–	4515	0.982	0.948	1.017	0.3106
*APOE* ɛ4 allele	Carrier	1642	1.715	1.400	2.101	<0.001*

Number of events: 413; global *P*-value (log-rank): 5.7915 × 10^−155^. Akaike information criterion: 4929.89; Concordance Index: 0.85. Hazards ratios calculated for subgroups with discrete categories used the following reference groups as comparators: no NPS (NPS group); MCI (cognitive status); female (sex); non-carrier (APOE ɛ4 allele). AD = Alzheimer’s disease; CI = confidence interval; MBI = mild behavioural impairment; MCI = mild cognitive impairment; NC = normal cognition; NPS = neuropsychiatric symptoms; TDP-43 = transactive response DNA-binding protein 43.

^*^ *P* < 0.05.

### Exploratory analyses

In the first exploratory analysis (Table 4), MBI tended to associate with post-mortem AD pathology, whether in isolation or with TDP-43/LBD co-pathology. With the exception of the AD+, LBD+, TDP+ outcome, non-MBI NPS was not associated with a higher likelihood of any pathological profile.

**Table 4:** Exploratory analysis of baseline NPS status (predictor) with post-mortem pathologies (outcome)

NPS status	AD+ (*n* = 294)	LBD+ (*n* = 76)	TDP-43+ (*n* = 60)	AD+TDP-43+ (*n* = 45)	AD+LBD+ (*n* = 128)	LBD+TDP-43+ (*n* = 20)	AD+LBD+TDP-43+ (*n* = 55)
No NPS (reference)	1.00	1.00	1.00	1.00	1.00	1.00	1.00
Non-MBI NPS	1.285 (0.195)	1.185 (0.290)	1.044 (0.335)	1.072 (0.414)	1.066 (0.256)	0.845 (0.557)	1.930* (0.355)
MBI+	1.565* (0.238)	0.535 (0.457)	0.847 (0.418)	2.183* (0.411)	1.214 (0.297)	0.518 (0.806)	2.021* (0.402)

Results are presented as odds ratios (standard error) for each NPS status relative to the no-NPS group, and were determined using a multinomial logistic regression with no pathologies (i.e. AD−, LBD−, TDP−) as the reference group was run adjusting for age, sex, education, cognitive status (MCI versus NC), time to death and *APOE* ɛ4 carrier status. AD+ = Alzheimer’s disease present; LBD+ = Lewy body disease present; MBI+ = mild behavioural impairment present; MCI = mild cognitive impairment; NC = normal cognition; NPS = neuropsychiatric symptoms; TDP-43+ = transactive response DNA-binding protein 43 present.

^*^ *P* < 0.1.

**Figure 4:**
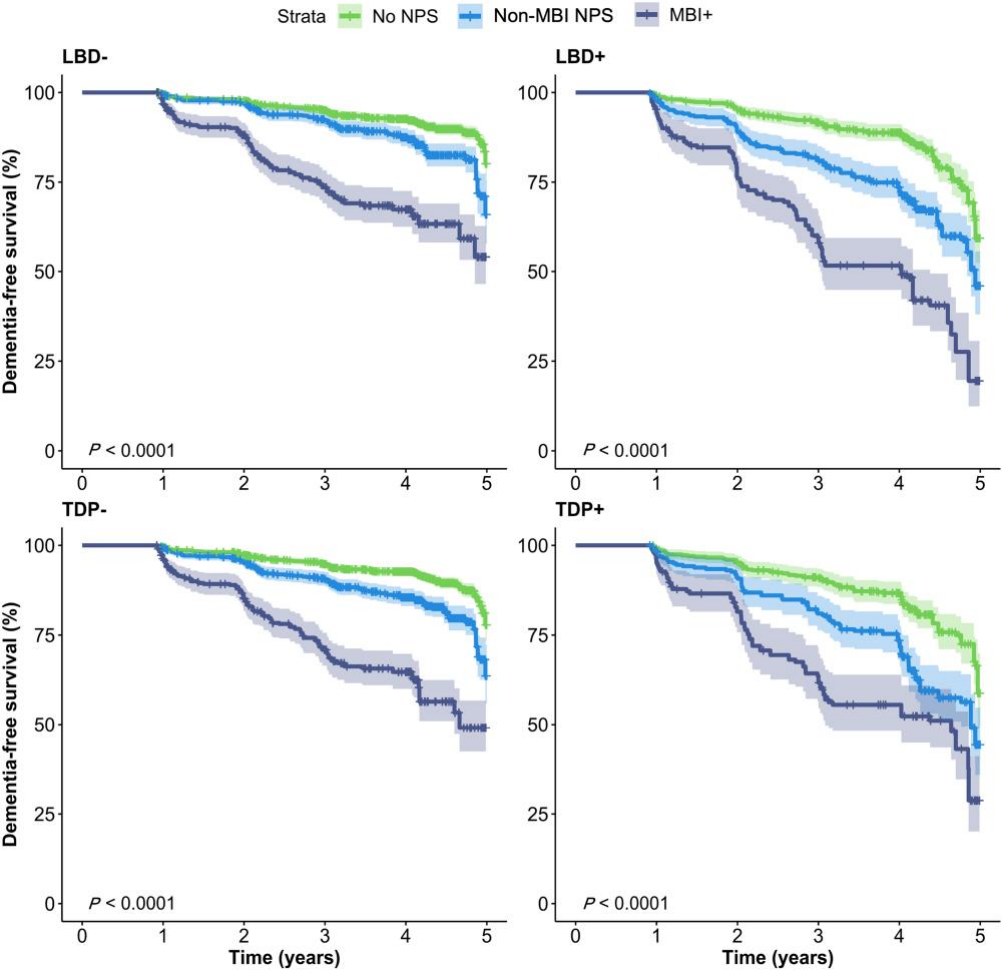
**Kaplan–Meier curve for 5-year progression to AD dementia, stratified by NPS status and presence/absence of proteinopathies.** LBD/TDP+ participants had a greater rate of incident AD dementia, although the differences were not significant from negative participants. When LBD pathology was stratified by regional presence, a significant moderation was seen for limbic LBDs. Note that participant strata between LBD and TDP groups are not mutually exclusive. AD = Alzheimer’s disease; LBD = Lewy body disease; MBI = mild behavioural impairment; NPS = neuropsychiatric symptoms; TDP-43 = transactive response DNA-binding protein 43.

The results of the second exploratory analysis revealed that the presence of limbic LBD significantly moderated the association between MBI and AD dementia within 5 years (*P* = 0.0319). For participants with limbic LBD, being MBI+ was associated with a 4.64-fold greater progression rate to AD dementia (hazard ratio: 4.64, CI: 2.05–10.50, *P* < 0.001); whereas for the MBI+ group without limbic LBD, there was a 1.87-fold progression rate (hazard ratio: 1.87, CI: 1.46–2.40, *P* < 0.001). No other regional LBD pathology, nor TPD-43 status, had any associations. Kaplan–Meier curves for each stratum are visualized in Fig. 4.

## Discussion

In this study, we examined the relationship between three common dementia-linked pathologies and NPS status in the 5 years preceding death. In this sample of older adults, dementia-free at baseline, we found that post-mortem AD pathology, but not LBD or TDP-43 co-pathology, had a greater likelihood of being MBI+ up to 5 years before death versus comparator NPS groups. In comparison to those without NPS, MBI+ participants progressed to AD dementia twice as fast. Limbic LBDs, but no other co-pathology, appeared to moderate the progression rate to AD dementia in those with MBI.

In line with the NIA-AA ATN framework, various lines of evidence have linked MBI with core AD pathology, whether at NIA-AA disease Stage 2 (encompassing NC and subjective cognitive decline) or Stage 3 (MCI). The studies exploring MBI versus conventionally identified psychiatric symptomatology, no MBI or no NPS have consistently found greater associations with AD for the NPS that are later-life emergent and persistent, i.e. MBI. Operationalizing MBI with these core criteria has face validity, given that 30% of persons who develop AD will develop NPS prior to cognitive impairment^5^ and that persistent NPS (versus transient or reactive symptoms) increase specificity that the identified NPS do indeed represent neurodegenerative disease rather than a reaction to life stressors, change or other psychiatric syndromes.^20,48,49^ These multi-modal MBI studies have found differences in CSF amyloid-β,^29^ plasma amyloid-β,^21^ β-amyloid PET,^27^ plasma p-tau181,^25^ tau PET,^30,50^ neuropathology^23^ and both imaging and biofluid markers of neurodegeneration.^31,32^

A longstanding question in dementia research is whether NPS have specific pathological substrates or are common across multiple diseases.^10,12,13^ However, here and previously, it has been shown that when NPS are operationalized following MBI criteria, an AD-specific association is observed; a further consideration that requires substantial study concerns the potential contribution of vascular pathology.^23-25^ This, alongside other common neuropathologies, such as tau, would help to elucidate whether certain NPS are disease-specific manifestations, and conversely, whether any given combination of disease processes is associated with a unique NPS profile.

The results of the case–control analysis showed that autopsy-confirmed AD was associated with a greater likelihood of having MBI in 5 years before death, but not non-MBI NPS. There were no associations seen for any co-pathology with MBI or non-MBI NPS. These findings suggest that development of MBI might be a specific marker for AD over other pathologies. Furthermore, the co-pathology sub-analysis among AD+ individuals reinforces these findings, demonstrating that having concomitant LBD and TDP-43 pathology does not increase the likelihood of having MBI or even any NPS. Although some studies point to the additive effect of LBD pathology on symptomatology in patients with ADNC, emerging literature suggests that behavioural symptoms might correlate better with AD rather than LBD pathology,^36,51^ with the likely exception of hallucinations.^52,53^ This might be attributable to the diffuse neuroanatomical involvement of AD rather than the comparatively narrow distribution of early-stage LBD pathology. Comorbid TDP-43 pathology has been shown to contribute to greater cognitive decline, but our results and previous work fail to demonstrate a similar link to NPS.^12,54^ Despite the high prevalence of co-pathologies in AD, the lack of any additional association between LBD or TDP-43 and MBI suggests that in individuals at risk of AD dementia, NPS as operationalized by MBI criteria might be a more specific correlate of ADNC. A potential explanation for this finding might relate to symptom fluctuations in dementia with Lewy bodies (DLB), such that NPS are less stable than in AD.^35^ These differences in stability might underscore the specificity of MBI for AD in multiple studies, given that symptom persistence is a cardinal MBI criterion conferring substantially greater specificity. Thus, NPS identified as MBI might represent an important manifestation of AD proteinopathy. However, in other datasets, MBI has been linked to Parkinson’s disease, also a synucleinopathy.^55,56^ Nonetheless, further study is required to address this novel finding.

Our finding from the sub-analysis is notable. In AD− individuals (‘No’ or ‘Low’ ADNC scores), neither TDP-43 nor LBD pathology was associated with MBI, in contrast to previous studies, suggesting that the link between co-pathologies and NPS is stronger in those earlier in the AD course.^12^ The similar trend we observed in AD+ individuals has been reported before in studies examining either concomitant LBD or TDP-43 pathology.^12,51,54^ Our results also build on another study showing that greater ADNC was linked to further NPS, but this study did not examine whether comorbid TDP-43 (associated with greater ADNC) had any further associations.^57^

Earlier research highlights NPS as having an independent association with AD pathology; specifically, neurofibrillary tangle burden.^58-60^ One study showed that NACC dementia participants with psychosis were often misdiagnosed with DLB, reflecting a potential misattribution of NPS to LBD-related dementias when NPS were likely to be a manifestation of AD.^52^ In another longitudinal study among dementia progressors with symptoms captured by MBI domain of psychosis (NPS more often ascribed to the DLB prodrome), 66.7% had clinician-diagnosed AD, in comparison to 10.0% for DLB and 0% for behavioural-variant frontotemporal dementia.^24^ In a similar study of participants showing symptoms from the MBI domain of apathy, 80.9% of dementia progressors had AD, 5.0% DLB and 4.3% behavioural-variant frontotemporal dementia. Finally, in a comparable study of MBI affective symptoms, 85.5% of progressors had AD, 3.7% DLB and 2.5% behavioural-variant frontotemporal dementia.^61^ Here, we demonstrate a comparable trend with neuropathological findings.

In comparison to the overall prevalence of each proteinopathy, a greater proportion of those with MBI were AD+ and had approximately equivalent levels of LBD and TDP-43 positivity. Among MBI+ individuals, the largest proportion had only AD pathology, in comparison to the other groups, where the largest category was no pathology. Interestingly, there was an ordinal relationship between post-mortem pathology and NPS status, wherein those with no NPS had the largest proportion of no pathology, followed by the non-MBI NPS group, and least of all, the MBI group, supporting the notion of MBI as being an AD-related phenomenon. Overall, these results suggest that those with MBI are often AD+, yet other pathologies are not any more prevalent in those with MBI. A larger proportion of those with MBI had multiple pathologies, potentially reflective of shared aetiological mechanisms between proteinopathies and/or as a consequence of a greater vulnerability to age-associated neuropathology.^62,63^

The survival analysis highlighted the utility of MBI as a prognostic marker for AD dementia. As we have shown, AD pathology, but not other pathologies, is linked to a greater likelihood of MBI before death. The results here suggest that in advance of dementia, MBI is associated with a more rapid progression to AD dementia. Importantly, neither these models nor the case–control ones showed any associations with non-MBI NPS. Thus, NPS on their own might not be a robust marker for AD, but when operationalized with MBI criteria, these symptoms become informative of AD risk. Although cognitive status was not a significant modifier of progression to dementia, the greater hazard ratio for incident dementia seen in the NC group is noteworthy. In previous studies, similar results have been found,^24,61,64^ demonstrating the relevance of behavioural symptoms in dementia prognostication, especially when no cognitive changes are observed. In such cases, a primarily behavioural presentation might not trigger a dementia work-up in clinic, instead leading to the misdiagnosis of a psychiatric prodrome to dementia as a psychiatric condition.^2,4,15^ Building on the NIA-AA neuropathological guidelines, which state that in those with cognitive impairment, the presence of intermediate or high ADNC is an ‘adequate explanation’ of symptoms,^41^ our results suggest that ADNC should also be considered as a primary contributor to MBI symptoms. Failure to acknowledge NPS as a part of a dementia prodrome has negative consequences both for those receiving inappropriate treatment for a misdiagnosed psychiatric condition and for early AD dementia detection and treatment trials relying solely on cognitive changes to select at-risk groups.^65^ With MBI, a subset of individuals can be selected from those showing NPS and identified for having a greater likelihood of progression to AD dementia.

With the diagnosis of dementia commonly being made in older age, an individual might have a primary pathology considered to be the main contributor to symptoms and yet potentially also harbour several other disease- and age-associated co-pathologies.^66^ In a clinicopathological study among community-dwelling participants, a majority of those who received a clinician diagnosis of dementia had more than one pathology identified at autopsy.^67^ With the advent of disease-modifying therapeutics directed at specific proteinopathies, failure to consider the presence of comorbid pathology might explain why a treatment does not appear to have clinical benefit.^63,68^ In the current treatment paradigm, clinicians should know the underlying pathological profile of a patient’s symptomatology and use that information to guide treatment. Thus, it is important to determine whether preclinical/prodromal AD is attributable to AD pathology or caused by the presence of co-pathologies recapitulating behavioural symptoms (e.g. TDP-43 in behavioural-variant frontotemporal dementia).^3,12^ If MBI is indeed specific to AD neuropathology, as our work suggests, then its implementation in clinical trials and practice would be of great aid in enriching target treatment groups with AD.^3^

Our study has a number of important limitations. For NPS measurement, we relied on the NPI-Q to capture symptomatology, implementing a published algorithm to operationalize MBI. However, the NPI-Q was developed for measurement of NPS in dementia and might lack sensitivity for detection of more subtle preclinical symptomatology or symptoms not overtly associated with dementia. To improve specificity, we included the stipulation of symptom persistence at more than two-thirds of visits, a validated approach to improve specificity.^46^ Additionally, the reliance of NPI-Q on informant reports poses a minor limitation, given its validation for use in dementia, in addition to the role of informant characteristics on accuracy of NPS reporting.^69^ Another shortcoming with our study was how we operationalized pathology; dichotomizing participants fails to account for the progressive nature of neurodegenerative disease. To address this, we conducted further analyses by stratifying participants by differential regional presence of LBDs but found no associations with higher likelihood of having NPS before death. Furthermore, we did not examine the associations of MBI symptom domains with any outcomes; these limitations all represent future directions for further research. Finally, NACC participants are not completely representative of the general clinical population owing to the referral/volunteer-based design of the study and geographical challenges in attending ADRCs. Future studies representative of a diverse clinical population are needed to shed further light on the value of the MBI construct.

## Conclusion

Taken together, these results add to the evidence pointing towards NPS as a core feature of preclinical and prodromal AD. In addition to changes in cognition, research and clinical frameworks should account for behavioural change in the staging of AD. In the 2018 NIA-AA framework, for clinical staging MBI is included in Stage 2. Our results, and others, support keeping new-onset persistent behavioural symptoms in the revised NIA-AA clinical framework. Furthermore, the inclusion of MBI in Stage 3 AD should also be considered, given the diagnostic and prognostic importance of later-life emergent NPS in MCI.

## Data Availability

All NACC data are freely available to researchers.
